# Building Blocks for the Development of a Self-Consistent Electromagnetic Field Theory of Consciousness

**DOI:** 10.3389/fnhum.2021.723415

**Published:** 2021-09-28

**Authors:** Joachim Keppler

**Affiliations:** Department of Consciousness Research, DIWISS, Roth, Germany

**Keywords:** consciousness, electromagnetic field theory, quantum electrodynamics, zero-point field, phase transitions, coherent states, resonance, neurotransmitters

## Abstract

The goal of this work is to compile the basic components for the construction of an electromagnetic field theory of consciousness that meets the standards of a fundamental theory. An essential cornerstone of the conceptual framework is the vacuum state of quantum electrodynamics which, contrary to the classical notion of the vacuum, can be viewed as a vibrant ocean of energy, termed zero-point field (ZPF). Being the fundamental substrate mediating the electromagnetic force, the ubiquitous ZPF constitutes the ultimate bedrock of all electromagnetic phenomena. In particular, resonant interaction with the ZPF is critical for understanding rapidly forming, long-range coherent activity patterns that are characteristic of brain dynamics. Assuming that the entire phenomenal color palette is rooted in the vibrational spectrum of the ZPF and that each normal mode of the ZPF is associated with an elementary shade of consciousness, it stands to reason that conscious states are caused by the coupling of the brain to a particular set of normal modes selectively filtered from the full frequency spectrum of the ZPF. From this perspective, the brain is postulated to function as a resonant oscillator that couples to a specific range of ZPF modes, using these modes as a keyboard for the composition of an enormous variety of phenomenal states. Theoretical considerations suggest that the brain-ZPF interface is controlled by altering the concentrations of neurotransmitters, placing the detailed study of the neurotransmitter-ZPF interaction at the center of future research activities.

## Introduction

As is typical of the spirit of discovery, those who are involved in consciousness research are driven by the ambition to develop the most comprehensive theory possible of their central research subject. Therefore, our efforts are directed toward the goal of constructing a fundamental theory of consciousness (TOC) that we expect to reveal the nature of phenomenal qualities (qualia), incorporate qualia seamlessly into the scientific framework, explain the relationship between phenomenal states and physical states, and correctly predict the phenomenal state of any system given its physical state.

In the following, arguments will be advanced that TOC candidates must establish a link to the fundamental theories of physics, particularly to the fundamental theory of electromagnetism. Building on this groundwork, the purpose of this article is to discuss the main aspects and pitfalls that have to be considered when constructing an electromagnetic (EM, or alternatively em) field TOC, to give an idea of a promising direction of thought with regard to an EM basis of conscious states, and to briefly outline future directions of research on the way to a full-fledged TOC. The main focus here lies on addressing fundamental conceptual issues with the aim of surmounting obstacles and identifying opportunities for the advancement of the research field.

## Linking Consciousness to the Foundations of Physics

One of the essential requirements for the development of a TOC consists in achieving a seamless integration of qualia into the edifice of science. In view of the fact that the phenomenal properties that constitute our mental inner world differ significantly from the properties we use to describe the physical world, it has long been pointed out that the prevalent variants of physicalism are fraught with explanatory gaps (Levine, 1983; Chalmers, 1995, 1996; Nagel, 2012). In the following, two of these gaps will be referred to as the cosmic latecomer problem and the demarcation problem. The *cosmic latecomer problem* arises in all approaches that consider conscious states as a product of cosmic evolution. According to such theories, consciousness awakens as soon as the structural conditions, the organizational principles, or the processes characterizing a physical system exceed a critical level of complexity, so that conscious states are assumed to emerge from or to be identical with certain activity patterns of sufficiently complex systems, as is the case for the brains of highly developed living beings (Edelman, 1989, 2003; Tononi and Edelman, 1998; Seth et al., 2006). This way of thinking, however, results in an ontological discontinuity and leaves its critics with the open questions of how, with the first-time generation of such activity patterns in a sufficiently developed brain, consciousness could appear in a previously insentient universe and what it is about these patterns that “suddenly switches consciousness on” (Velmans, 2007). Furthermore, in the absence of a properly specified basis for consciousness, the proponents of the latecomer hypothesis are confronted with the *demarcation problem*, which consists in the challenge of explaining why a certain level of organization should be associated with subjective awareness and what exactly distinguishes activity patterns that are accompanied by conscious experiences from those patterns that are devoid of any phenomenal qualities (Seager, 1999).

To get around the cosmic latecomer problem, it seems reasonable to resort to the fundamental description level of the physical world and to strive to establish a relationship between consciousness and the foundational entities and mechanisms that show up in the cosmic blueprint. This blueprint is reflected in the standard model of particle physics and cosmology (Cottingham and Greenwood, 2007; Rich, 2010), and even though there are unresolved issues and indications of physics beyond the standard model (Klapdor-Kleingrothaus et al., 2011; Nagashima, 2014; Bambi and Dolgov, 2016), the conceptual bedrock this model relies on provides a deep understanding of our universe.

Among the fundamental forces incorporated in the standard model, the EM interaction is of central importance for the development of a TOC, as it is by far the dominant force on the length scale of biological organisms. However, while the EM interaction has the status of a fundamental interaction, classical electrodynamics, being incomplete, does not have the status of a fundamental theory (Frisch, 2005). Rather, the theory that accounts for all the subtleties of the EM interaction is quantum electrodynamics (QED) which, contrary to the classical notion of the vacuum, includes a vacuum state “with a rich structure, full of energy and potentialities” (Kuhlmann et al., 2002). According to a recent school of thought, subsumed under the umbrella of stochastic electrodynamics, which strives to find a consistent description of reality and to derive the formalism of QED from this description, the vacuum state is interpreted as an omnipresent EM background field, termed *zero-point field* (ZPF), characterized by a spectrum of uncorrelated normal modes that satisfy a unique spectral energy density (Marshall, 1963, 1965; Boyer, 1975; De la Peña and Cetto, 1994, 1995, 2001, 2006; De la Peña et al., 2009, 2015). From this perspective, it is the ZPF that acts as the fundamental substrate of the EM force and, therefore, constitutes the ultimate basis of the EM interaction. The key insight, then, is that *all EM phenomena are mediated by the ubiquitous ZPF*. Thus, while the classical theory offers, for example, just a suitable calculation formula for the electric potential of a given configuration of charges, the complete theory reveals the underlying mechanisms and explains how this potential originates from the charges being embedded in the ZPF (Cohen-Tannoudji et al., 1997).

Apart from disclosing fundamental mechanisms, the relevance of QED to consciousness research rests on being pivotal to the understanding of complex dynamical systems. More precisely, a proper description of the dynamics of biological systems, particularly the dynamics of the brain that is characterized by long-range coherence and rapidly forming activity patterns resulting from *second-order phase transitions*, necessitates the theoretical foundations of QED and the presence of the ZPF (Del Giudice et al., 1985, 2005; Freeman and Vitiello, 2006). In such *macroscopic quantum phenomena*, the collective behavior of the system components is caused by their resonant coupling to a system-specific set of relevant normal modes selectively filtered from the full frequency spectrum of the ZPF (De la Peña and Cetto, 2001; De la Peña et al., 2015). In the event that the resonant system-ZPF interaction leads to the formation of a *transiently stable attractor*, a partial organization of the local field ensues in such a way that the relevant ZPF modes become highly correlated (De la Peña and Cetto, 2006; De la Peña et al., 2009), meaning that “the orchestration of an attractor requires the initially chaotic ZPF to change over to a partially ordered state” (Keppler, 2018).

The long-range order phenomena characteristic of neural activity patterns cannot be accounted for on the basis of classical physics since a viable mechanism governing the collective cooperation of system components is missing (Freeman and Vitiello, 2006). Rather, theoretical considerations on the foundations of quantum physics reveal that the emergence of coherent structures can be attributed to the unique properties of the ZPF (De la Peña and Cetto, 2001; De la Peña et al., 2009), the essential finding being that this field operates as a “formative agent behind the scenes” that has no equivalent in classical physics (Keppler, 2016).

Taken together, the above findings suggest that the ZPF could be the key entity for the development of a TOC. Before exploring this idea in detail, let us first take a brief look at classical EM approaches to consciousness.

## Looking at Classical Electromagnetic Field Theories of Consciousness

In order to highlight the main obstacles of classical EM field theories, the analysis will be confined to the most prominent representatives of this branch and their basic premises (Pockett, 2000, 2002, 2012; John, 2001, 2002; McFadden, 2002a,b, 2013, 2020; Fingelkurts et al., 2009, 2010, 2013). For a more extensive discussion, see Jones (2013). While all representatives share the assumption that the brain’s EM field is the substrate of consciousness, the various approaches differ significantly in their conceptual underpinnings. One of the theories holds the view that “conscious experiences are identical with certain spatial EM patterns generated by neural activity” (Pockett, 2012). According to other theories, consciousness is thought of as an “emergent property of sufficiently organized energy” (John, 2002), understood as the “inner experience of information… encoded in the brain’s em field” (McFadden, 2002b), or assumed to be related to the “nested hierarchy of spatiotemporal patterns of 3D electromagnetic fields produced by neuronal assemblies” (Fingelkurts et al., 2013). This means that with respect to the psychophysical nexus, i.e., regarding the relationship between phenomenal states and physical states, the theories represent markedly different positions: Pockett’s approach is a variant of identity theory, John’s approach a variant of emergentism, McFadden’s approach is based on the double-aspect theory of information (Chalmers, 1995, 1996), whereas Fingelkurts et al. (2013) defend supervenience through isomorphism.

Ultimately, independent of the position, all these approaches consider consciousness as a cosmic latecomer and face the demarcation problem which consists in the challenge of presenting a convincing model that explains what exactly distinguishes EM field patterns which are accompanied by conscious experiences from those patterns or configurations to which phenomenal zero-states are to be assigned. In this regard, the proponents of classical EM field theories are aware of the need for a threshold criterion, emphasizing that conscious experiences are limited to “certain spatial EM patterns” (Pockett, 2012) or “sufficiently organized energy” (John, 2002), or pointing out that the “minimal characteristic of an em field to qualify as conscious must surely be that is possesses sufficient complexity” (McFadden, 2020). However, it remains largely open what counts as *sufficiently complex* to exceed the threshold of consciousness. Proposed solutions to make the criterion more tangible consist in imposing constraints on the EM field in such a way that the defining feature of conscious field configurations is hypothesized to reside in very specific spatial patterns of local field potentials (Pockett, 2012), or that the conscious component of the brain’s EM field is linked to the capability of “initiating motor actions” (McFadden, 2002b) or “transferring thoughts to another conscious being” (McFadden, 2020). Yet, proposals of this kind raise the question as to why any constraint on a physical field, whether configurational, functional, or communicational, should mark the dividing line between conscious and unconscious states.

In essence, all variants of classical EM field theories of consciousness encounter an explanatory gap that can be traced back to the missing link to the fundamental theory of the EM interaction and the absence of a comprehensible mechanism which endows certain field configurations, or the brain processes that give rise to these field configurations, with phenomenal qualities. In order to find a way out of the quandary, we will now return to the conceptual basis of QED.

## Toward a Self-Consistent Electromagnetic Field Theory of Consciousness

**FIGURE 1:**
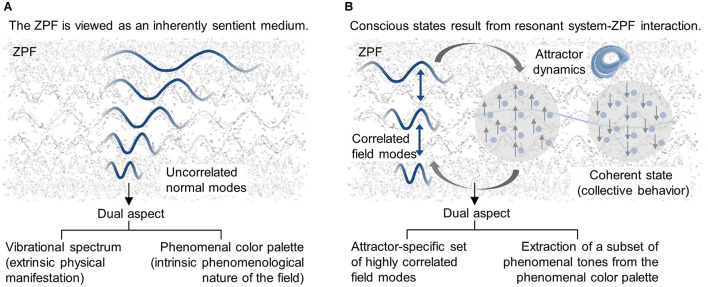
Toward a self-consistent EM field theory of consciousness. **(A)** The zero-point field (ZPF) is assumed to be a foundational dual-aspect component of the cosmos, the extrinsic manifestation of which is physical in nature and the intrinsic manifestation of which is phenomenological in nature. **(B)** It is postulated that conscious systems employ a universal mechanism through which they tap into the phenomenal color palette predetermined by the ZPF. According to this mechanism, conscious states are caused by resonant system-ZPF interaction, as a result of which the system falls into an attractor and the relevant ZPF modes involved in the interaction combine into an attractor-specific set of highly correlated field modes.

A promising approach to the scientific understanding of conscious systems is predicated on the notion that phenomenal qualities are irreducible features of reality and that the ZPF is the substrate of consciousness (Keppler, 2012, 2013, 2016, 2018, 2020; Shani and Keppler, 2018; Keppler and Shani, 2020). In concrete terms, it is proposed that the ZPF is “an *inherently sentient medium*”, i.e., “a foundational *dual-aspect* component of the cosmos, the extrinsic appearance of which is physical in nature and the intrinsic manifestation of which is phenomenological in nature” (Keppler and Shani, 2020), implying that the entire color palette of consciousness is rooted in the vibrational spectrum of the ZPF and that each normal mode is associated with an elementary shade of consciousness (see Figure 1A). Considering its disordered ground state, the ZPF can thus be conceived as “a *formless sea of consciousness* or unstructured ocean of awareness that carries an enormous range of potentially available phenomenal nuances” (Shani and Keppler, 2018). To sum up, the ZPF is postulated to be a psychophysical “entity that embodies the principles of physics and at the same time contains within itself the phenomenological basis of ultimate reality” (Keppler and Shani, 2020). The inner structure of the ZPF is thereby arranged in field modes which reveal themselves physically as oscillations with specific frequencies and phenomenologically as shades of awareness.

Following this line of thought, it stands to reason that conscious systems employ a universal mechanism through which they tap into the phenomenal color palette predetermined by the ZPF. Recalling the previously outlined mechanism underlying macroscopic quantum phenomena, one is led to assume that “*the formation of transiently stable coherent states is an essential prerequisite for conscious awareness*” (Keppler, 2018). According to this mechanism, it is postulated that conscious states are caused by resonant system-ZPF interaction, as a result of which the system falls into an attractor and the relevant ZPF modes involved in the interaction combine into an attractor-specific set of highly correlated field modes (see Figure 1B). From this perspective, “a physical system acquires phenomenal properties by entering into a temporary liaison with the cosmic field of consciousness and extracting a subset of phenomenal tones from the spectrum of all phenomenal tones potentially present in the field” (Keppler and Shani, 2020), suggesting that “*a distinctive feature of conscious systems in comparison to non-conscious systems must be the capacity to modulate the omnipresent field of consciousness*” (ibid.). In vivid terms, then, conscious experiences are restricted to those systems that manage to play the keyboard of the ubiquitous field of consciousness.

Based on the presented approach, a clear dividing line can be drawn between conscious and non-conscious systems. Even though all types of systems are permeated by the ubiquitous ZPF and thus, from a phenomenological perspective, are surrounded by an ocean of potential, yet undifferentiated consciousness, the formation of concrete conscious states is confined to those systems that can dynamically interact with the ZPF, which requires the resonant coupling of the system to a set of ZPF modes. These dynamical properties are unique to quantum systems, whose coupling to the ZPF is reflected in long-range coherence and attractor formation. Importantly, “attractors manage the transition from potentiality to actuality,” implying that “a potential conscious state is actualized once an attractor is fully unfolded” and a corresponding attractor-specific modification of the ZPF arises (Shani and Keppler, 2018). In contrast, classical systems are not dynamically coupled to the ZPF, indicating that this kind of systems cannot access the ZPF’s immanent phenomenal color palette and therefore cannot engender concrete phenomenal states.

Crucially, the findings of neuroscience are fully compatible with this line of reasoning. More precisely, the body of empirical evidence indicates that a stream of consciousness is based on periodically recurring, highly synchronous neural activity (Desmedt and Tomberg, 1994; Rodriguez et al., 1999; Engel and Singer, 2001; Melloni et al., 2007; Doesburg et al., 2009; Gaillard et al., 2009; Singer, 2015). In particular, the studies of Freeman revealed that the neural correlates of conscious perception can be equated with large-scale patterns of coherent gamma-band activity that occur at theta rates and represent *attractors* in an attractor landscape (Freeman, 2004, 2005, 2007, 2009). The fact that the rapid synchronization of the large-scale patterns proceeds in the form of scale-free neuronal avalanches suggests that the attractor formations involve critical phenomena and *second-order phase transitions* (Beggs and Plenz, 2003; Kitzbichler et al., 2009; Cocchi et al., 2017), the rigorous explanation of which requires the framework of quantum field theory (Zinn-Justin, 1996; Freeman and Vitiello, 2006, 2007). These insights support the hypotheses that the orchestration of coherent neural activity patterns takes place via the ZPF and that the brain generates an individual stream of consciousness by periodically modulating the ZPF (Keppler, 2013, 2016, 2018, 2020).

In summary, the brain is postulated to function as a *resonant oscillator* that couples to a specific range of ZPF modes, using these modes as a keyboard for the composition of an enormous variety of conscious states. As far as the concept of resonance is concerned, the approach presented here shares commonalities with the theory of Hunt and Schooler (2019), according to which resonance-induced phase transitions underlie the formation of macro-conscious entities. The ZPF-based conceptual framework specifies the resonance mechanism in greater detail, sets the course for clear-cut future research projects (see the following section), and meets the key criteria to be imposed on a TOC candidate. In particular, it has explanatory power and respects the principles of parsimony and universality by spelling out how dynamical systems interacting with the ZPF gain “access to the ubiquitous substrate of consciousness” and “acquire both their physical properties and their phenomenal qualities by use of one and the same mechanism” (Keppler, 2018), the proposed modulation mechanism being “intelligible and completely transparent” (Keppler, 2020). This mechanism, which “is deeply rooted in the foundations of the universe”, results in “well-defined distinctive features between conscious and non-conscious systems as well as conscious and unconscious brain processes” (Keppler and Shani, 2020), thereby remedying the demarcation problem the classical approaches struggle with. Finally, it is worth mentioning that beyond the processes behind conscious perception, the presented approach also provides satisfactory interpretations of the neural correlates of self-referential conscious processes (Keppler, 2018) and episodic memory processes (Keppler, 2020).

To conclude this section, some notes are appropriate with regard to the positioning of the ZPF-based conceptual framework in the current theory landscape and the clarification of the differences to as well as the intersections with contemporary neuroscientific theories of consciousness, the most prominent representatives of which assume conscious awareness to be associated with a global workspace that connects and coordinates widely separated brain areas (Baars, 1988, 2005; Dehaene and Naccache, 2001; Dehaene et al., 2006), with synchronously firing coalitions of neurons (Crick and Koch, 1990, 2003), with a dynamic core corresponding to a functional cluster of neurons forming transiently stable activity patterns (Tononi and Edelman, 1998; Edelman, 2003), or with recurrent processing (Lamme, 2006). Overall, the ZPF-based approach leads to a reinterpretation and reassessment of the neural correlates to the effect that they should not be held responsible for the mysterious generation of consciousness but, rather, viewed as corollaries of a deeper mechanism by which neural cell assemblies couple to an omnipresent field of consciousness. In this scenario, the recurrent formation of transiently stable activity patterns displaying gamma synchrony indicates that streams of consciousness have their origin in the periodic modification of this field. From this perspective, the ZPF, which is accountable for the coordination of brain areas and the synchronization of brain activity, may be understood as *the truly global workspace* in which conscious processes unfold. As for the comparison of the ZPF-based approach with the integrated information theory (Tononi, 2004, 2008; Oizumi et al., 2014), the reader is referred to Keppler (2016), while a detailed discussion of the positioning of the approach in the field of panpsychism can be found in Shani and Keppler (2018).

## Looking Ahead to Future Research Avenues

The outlined modulation mechanism guides us to place the brain-ZPF interface at the center of future research activities. A suitable basis for the description of the phase transitions underlying the formation of coherent activity patterns can be found in the theory of *superradiant phase transitions* (Hepp and Lieb, 1973; Wang and Hioe, 1973). Such phase transitions arise when the interaction between an ensemble of molecules and the ZPF exceeds a critical coupling strength, which is precisely the case when selected ZPF modes are in resonance with the characteristic transition frequencies between molecular energy levels and the concentration (density) of molecules lies above a critical threshold (Preparata, 1995; Del Giudice et al., 2005). The wavelengths of the selected ZPF modes define the extent of the *coherence domain*, in the interior of which the molecules exhibit collective behavior, causing a decrease in the energy per molecule and energetically stabilizing the coherence domain against its environment (Preparata, 1995; Del Giudice et al., 2005). The presence of interfacial water can lead to an expansion of coherence domains and provides additional shielding from destructive thermal influences (Del Giudice et al., 2010, 2013).

**FIGURE 2:**
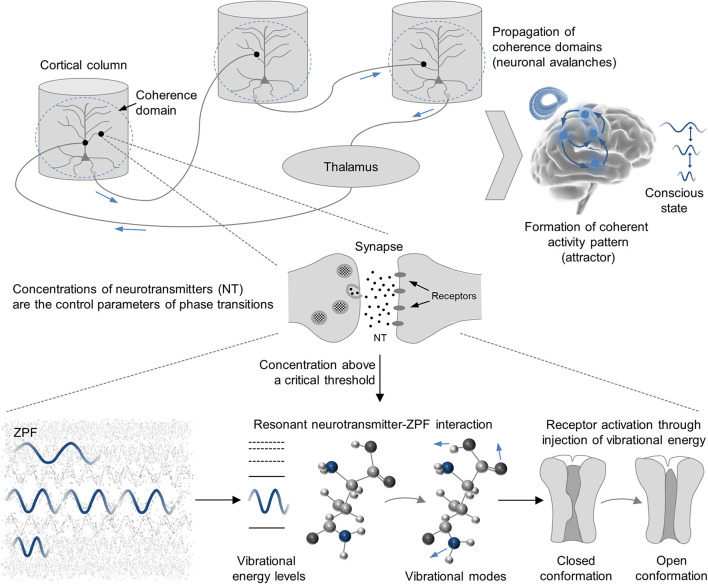
The neurophysiological mechanisms underlying conscious processes. It is postulated that the phase transitions underlying the formation of coherent activity patterns (attractors) are triggered by modulating the concentrations of neurotransmitters. When the concentration of neurotransmitter molecules lies above a critical threshold and selected ZPF modes are in resonance with the characteristic transition frequencies between molecular energy levels, receptor activations ensue that drive the emergence of neuronal avalanches. The set of selected ZPF modes that is involved in the formation and stabilization of an attractor determines the phenomenal properties of the conscious state.

Applying this theory to the brain, it is to be expected that neuromodulators control the observed phase transitions by regulating the concentrations of neurotransmitters (Chialvo, 2010), in agreement with the finding that the formation of neuronal avalanches and synchronized activity patterns depends on the density of neurotransmitters, such as the common glutamate and GABA receptor agonists, as well as neuromodulators, such as dopamine (Stewart and Plenz, 2006; Gireesh and Plenz, 2008). In conjunction with the notion that the receptor activations driving the emergence of neuronal avalanches are induced by specific vibrational modes of the participating agonists (Kubo et al., 2001, 2003), these insights point to the crucial role of neurotransmitter-ZPF interactions in the generation of conscious states (for an illustration of the mechanism, see Figure 2). As cortical areas differ in their receptor fingerprints (Zilles and Palomero-Gallagher, 2017), the characteristic neurotransmitter-receptor profile of an area should determine the set of selected ZPF modes and, consequently, the spectrum of accessible phenomenal tones.

The focus of future research efforts must be to understand the proposed mechanism more precisely from a theoretical point of view and to substantiate the mechanism experimentally. The corresponding research program can be divided into physical, neurochemical, neurophysiological, and phenomenological avenues of collecting evidence, taking into account the different organizational levels and dimensions of conscious processes.

At the basic physical level, corroborating empirical evidence for the proposed mechanism can be obtained by demonstrating the modulation of the ZPF during conscious states, which requires the measurement of phase correlations between ZPF modes. In recent years, the experimental basis has been developed to perform direct measurements of field correlations of the electromagnetic vacuum state (Riek et al., 2015; Benea-Chelmus et al., 2019), so that these methods may be applicable to the detailed investigation of brain-induced ZPF modulations in the foreseeable future.

The neurochemical level is primarily concerned with exploring the neurotransmitter-ZPF interface. For this purpose, following the conceptual groundwork laid by Preparata (1995), the formalism of QED needs to be applied to derive equations describing the interaction between ZPF modes and vibrational modes of the neurotransmitter molecules. Using these equations, the critical concentrations required for resonant neurotransmitter-ZPF coupling and thus for the induction of phase transitions can be calculated. Comparing the calculated critical neurotransmitter concentrations with experimentally determined concentrations will allow conclusions to be drawn about whether neurotransmitter-ZPF interactions play a crucial role in the generation of the cortical phase transitions that occur when conscious states are formed.

To get to the neurophysiological level, the theoretical apparatus may be used to perform simulations aimed at studying the macroscopic consequences of neurotransmitter-ZPF interactions and making theoretical predictions about the dynamical characteristics of the neural correlates of consciousness (NCC). Based on these predictions, advanced analysis techniques can be employed to screen the available data for signatures that, in addition to the evidence already presented in the previous section, lend further support to the notion of the ZPF being instrumental in the formation of the activity patterns constituting the NCC.

Both the level of neurotransmitters and the macroscopic system level provide the opportunity to collect supplementary empirical evidence for the existence of the brain-ZPF interaction mechanism, resulting from the prediction that the postulated “phase transitions are expected to be accompanied by collective emissions of photons” (Keppler, 2020). The experimental methods for detecting such phenomena, termed ultraweak photon emissions or biophoton emissions, are well developed (Popp et al., 1994; Cohen and Popp, 1997; Popp, 2003). At the neurotransmitter level, photon emissions should be triggered upon exceeding a critical density, and indeed studies using a biophoton imaging system revealed that above a critical threshold concentration glutamate causes a significant elevation of biophotonic activity (Tang and Dai, 2014). At the macroscopic level, characteristic photon pulses should follow the theta cycle, due to the finding that during processes of conscious perception the phase transitions that induce the formation of coherent activity patterns occur at theta rates. There is preliminary experimental evidence to support this hypothesis (Kobayashi et al., 1999), just as there are indications that biophotonic activity depends on the state of consciousness (Van Wijk and Van Wijk, 2005; Van Wijk et al., 2005). Future studies must be designed to allow comparison of measured photon signals with theoretical expectations with the goal of reconstructing the modification of the ZPF associated with a particular conscious state.

Once sufficient expertise has been accumulated in the methods outlined above for measuring or reconstructing the physical concomitants of conscious states, one can venture into the exploration of the phenomenological structure of the ZPF. This project involves guiding subjects through a variety of phenomenal states, determining the modified ZPF state associated with each phenomenal state, and systematically calibrating the determined ZPF states based on the first-person accounts (Keppler, 2016), leading to the “derivation of psychophysical mapping rules between particular qualia and particular sets of phase-locked ZPF modes” (Shani and Keppler, 2018).

In case of corroboration, the conceptual framework presented here would lead to a refinement of previously formulated ideas, according to which the activity of coherently oscillating cell assemblies is orchestrated by synaptic input to the dendrites of cortical pyramidal cells (Nunez and Srinivasan, 2006), neurotransmitters might change the resonance properties of cortical areas by altering their coupling strengths to “synaptic action fields” (ibid.), and oscillating activity in the “synaptodendritic web” is assumed to play an important role in conscious processes (Pribram and Meade, 1999).

In conclusion, the presented approach is based on the position that, in order to avoid explanatory gaps, a TOC candidate must build a bridge to the fundamental theories of physics and that particularly QED, being the fundamental theory of the electromagnetic interaction and being crucial for the understanding of complex dynamical systems, is of central importance for the development of a TOC. More specifically, the ZPF is assumed to be a foundational psychophysical component of the cosmos, implying that each normal mode of the ZPF is associated with an elementary shade of consciousness. It is argued that the brain generates conscious states by resonant coupling to ZPF modes, setting the course for a number of novel research projects on the study of consciousness.

## Data Availability Statement

The original contributions presented in the study are included in the article/supplementary material, further inquiries can be directed to the corresponding author.

## Author Contributions

The author confirms being the sole contributor of this work and has approved it for publication.

## Conflict of Interest

The author declares that the research was conducted in the absence of any commercial or financial relationships that could be construed as a potential conflict of interest.

## Publisher’s Note

All claims expressed in this article are solely those of the authors and do not necessarily represent those of their affiliated organizations, or those of the publisher, the editors and the reviewers. Any product that may be evaluated in this article, or claim that may be made by its manufacturer, is not guaranteed or endorsed by the publisher.
